# Influence of Geographical and Seasonal Variations on Carbazole Alkaloids Distribution in *Murraya koenigii*: Deciding Factor of Its *In Vitro* and *In Vivo* Efficacies against Cancer Cells

**DOI:** 10.1155/2020/7821913

**Published:** 2020-02-11

**Authors:** Eswara Murali Satyavarapu, Prasun Kumar Sinha, Chitra Mandal

**Affiliations:** Cancer Biology and Inflammatory Disorder Division, Council of Scientific and Industrial Research-Indian Institute of Chemical Biology, 4, Raja S.C. Mallick Road, Kolkata 700032, India

## Abstract

*Murraya koenigii* is a well-known Indian medicinal herb, and a carbazole alkaloid (mahanine) from this plant causes apoptosis in cancer cells. Here, we investigated how seasonal and geographical variations influence carbazole alkaloids composition and medicinal property of this plant against cancer cells *in vitro* and *in vivo*. Leaflets were collected from various places in different seasons for three years. A mahanine-enriched fraction (MEF) was prepared in two steps using ethanol and water. The best plant was selected based on the highest percent of mahanine. MEF prepared from leaflets of nine different locations showed a different concentration of identified markers (mahanine, mahanimbine, and koenimbine) which exhibited differential reduced metabolic activity against ovarian cancer, mahanine being the best. Our systematic study revealed that mahanine content was highest during September–December. Interestingly, MEF from southern part (tropical zone) exhibited 43 ± 2.5% mahanine compared to  2.7 ± 1.3% in northeastern part (subtropical zone) with five folds higher activity against PA1. Moreover, MEF reduced metabolic activity of sixteen cancer cell lines from nine different origins and significantly reduced tumor mass in lung and ovarian cancer xenograft models. Taken together, this is the first report demonstrating the marker's content in these leaflets is highly dependent on location/season. A positive correlation between biological activity and mahanine concentration was established in MEF. Such a comprehensive study suggests that the selection of location and suitable season for collection of any plant materials with biologically active stable markers in sufficient quantity play a decisive role in determining the fate of their medicinal property.

## 1. Introduction

Natural products, especially from plants, are important medicinal source for many diseases as documented in *Charaka Samhita*, a comprehensive text on ancient Indian medicine written during 200–400 B.C., known as “*Encyclopedia of Ayurveda*” [1]. India is considered to be the botanical garden of the world. A wide variety of plants with an array of medicinal properties has been reported [2]. However, the medicinal value of these plants depends upon several ecological factors which include climatic factors like rainfall, atmospheric humidity, wind, gases, temperature, and light; physiographic factors (altitude and sunlight); and biotic and abiotic factors (soil microorganisms and nutrients) [3]. Moreover, such ecological factors varied based on different seasons and diverse geographical regions. Therefore, it is worthwhile to prepare a detailed document for the optimization of the conditions for the collection of plant material and in-depth investigation for the best utilization of such herbal product for a specific disease.

Accordingly, we have carefully selected an edible plant, *Murraya koenigii* (L.) Spreng, commonly called as “curry leaves” or “Kari patta or Karuveppilai or Karivepaku” abundantly available throughout India, for such documentation. This herb belongs to the family Rutaceae, leaves are pinnate, with 11–21 leaflets, and each leaflet is 2–4 cm long and 1–2 cm broad. This plant has been reported for its traditional uses like diarrhea, indigestion, and nausea in *Charaka Samhita* [1]. In the last ten years, we have isolated and extensively characterized a biologically active carbazole alkaloid (mahanine) from the leaflets of this plant and established its anticancer activity against different cancer cells with various mutations [4]. Our extensive study also ascertained its role in controlling several cell survival pathways in leukemia, pancreatic, ovarian, glioblastoma, cervical, and colon cancers. We also observed that mahanine is a pro-oxidant molecule. It induced death receptor-mediated apoptosis in leukemia, acts as mTORC1/2 inhibitor in glioblastoma multiform [5] and regulation of hedgehog pathway [6] and, acts as Hsp90 inhibitor in pancreatic [7] and enhanced tumor suppressor proteins in colon cancers, inhibited autophagy and LC3-mediated anoikis in ovarian cancer [8]. Additionally, it showed a good synergistic effect with clinically approved drugs both in colon and cervical cancers. It also exhibited antileishmanial activity through immunomodulation [9].

Accordingly, we aimed to prepare a mahanine-enriched fraction (MEF) from *Murraya koenigii* leaflets. In search of the highest mahanine-containing plant, we performed an elaborate, systemic detailed comparative study to understand the relationship of seasonal and geographical variations of other molecules present in MEF including mahanine in the leaflets of *M. koenigii* and compared their biological activities. Accordingly, we have collected the leaflets from all over India having different climatic conditions, altitude, and sunlight and soil properties throughout the year.

Here, we are reporting a two-step protocol for the preparation of MEFs using only two edible solvents from leaflets collected during different seasons throughout India. Next, we identified and characterized three carbazole alkaloids (mahanine, mahanimbine, and koenimbine) from MEF and compared their reduced metabolic activity against two representative ovarian cancer cell lines (PA1 and OVCAR3) at different concentrations. We found that mahanine content in the leaflet was more in the southern part of India during September–December, which is directly proportional to the highest biological activity *in vitro*. MEF also exhibited reduced tumor mass in ovarian, lung cancer xenograft mice. To our knowledge, it is the first report of systematic detailed study to determine the best place and season to collect *M. koenigii* leaflets with the highest medicinal values to elevate the commercial value of this high-demand plant.

## 2. Materials and Methods

### 2.1. Cell Cultures

Human ovarian (PA1 and OVCAR3), lung (A549), glioblastoma (U87MG), pancreatic (MIAPaCa-2), colorectal (HCT116), cervical (Hela), melanoma (Sk-mel-28), and osteosarcoma (MG-63) cancer cell lines were purchased from NCCS cell repository, Pune.

Additionally, melanoma (ChaMel47), ovarian (SKOV3, OAW42, and UWB1.289+BRCA1), glioblastoma (U87MGvIII), and squamous oral (UPCI-SCC-084 and UPCI-SCC-131) carcinoma cell lines were kind gifts of Prof. Peter Walden, Charité-Universitätsmedizin, Berlin, Germany; Dr. SS Roy, CSIR-IICB; Dr. Frank Furnari, Ludwig Cancer Research Institute, US; Dr. Asima Mukhopadhyay, Tata Medical Center; and Dr. Susanta Roychoudhury, Saroj Gupta Cancer Center & Research Institute, Kolkata, respectively. All Cell lines were grown in complete medium (Table 1) supplemented with 10% FBS and 1% antibiotic-antimycotic at 37°C, 5% CO_2_.

### 2.2. Collection and Drying of Plant Material

“*Murraya koenigii* (L.) Spreng” belongs to the family Rutaceae (http://www.theplantlist.org) and is widely available all over India. Plant leaflets were procured throughout the year and also from east, west, north, south, and middle parts of India for three consecutive years. These were identified by Dr. Debabrata Maity, Department of Botany, Kolkata, where a voucher specimen was deposited (20033(CUH)). These fresh leaflets (1.0 kg) were washed thoroughly with portable water followed by distilled water and dried in a dust-free environment. The dried leaflets (0.2 kg) were grinded into small pieces.

### 2.3. Mahanine-Enriched Fraction (MEF)

Small pieces of dried leaflets (100 gram) were kept in distilled water (2.0 liters) at 50°C for four hours with stirring occasionally. The water portion was separated by filtration, and the remaining residue was dried (75 grams) for maceration with ethanol (1.5 liters) at ∼30°C for 72 hours. This ethanolic fraction was dried. The yield of MEF from 100 g of dry leaf powder was calculated and stored at −20°C in an airtight container.

### 2.4. HPLC Analysis of MEF

For quantification of markers by HPLC (Waters, 2487, Dual *λ* Absorbance UV Detector), MEF (500 *μ*g/ml) was dissolved in methanol and water (80 : 20) and filtered using 0.22 *µ*m syringe filter. The RP-C18 column (5 *μ*m, 250 × 4.6 mm, Waters, USA) was used. Sample (200 *μ*l) was injected and run in an isocratic solvent system with 1.0 ml/minute flow rate. Peaks were monitored using a UV detector at 254 nm. All peaks area and retention time (RT, minutes) were calculated using Empower 2 software. Similarly, HPLC fingerprints of all MEFs including water extract were compared. The external calibration method was used to quantify the markers in MEF using pure compounds (5–20 *μ*g) by HPLC [10].

### 2.5. Isolation and Characterization of Markers

MEF was packed on the silica column and eluted with petroleum ether and chloroform (100-0 : 0-100). Different fractions were monitored by TLC. Mahanine and mahanimbine fractions were separated by comparing with reference compounds which were previously isolated by our group [4]. Mahanine and mahanimbine were further purified by preparative TLC and HPLC using a reverse-phase C18 column to get ≥95% purity. Purification was confirmed by thin layer chromatography (TLC), high pressure liquuid chromatography (HPLC), and mass spectrometry (ESI-Q TOF instrument), and the structure was confirmed by ^1^H Nuclear magnetic resonance (NMR) and ^13^C NMR (Bruker 600 MHz and JEOL 400 MHz instruments) [4]. Koenimbine-containing fraction was washed thrice with methanol to yield pure compound and characterized similarly.

### 2.6. MTT Assay

MEF was dissolved in ethanol (10 mg/ml), and substocks were prepared with complete medium. The bioactivity of MEF, mahanine, mahanimbine, and koenimbine against cancer cell lines was determined separately by the MTT (3-(4,5-dimethylthiazol-2-yl)-2,5-diphenyltetrazolium bromide) assay. PA1 was harvested using trypsin and counted using the hemocytometer in the presence of trypan blue. Washed cells (4 × 10^3^ in 250 *μ*l/well) were placed in a 96-well plate and incubated for 1–2 hours. MEF (0–100 *μ*g/ml) was added to each well in triplicates and incubated for 48 h at 37°C. The medium was discarded, and MTT (dissolved in IMDM, 100 *μ*g/well) was added and incubated for 3 h at 37°C. Formazan crystals formed by the live cells were dissolved in DMSO. The intensity of the color was quantified at 550 nm in an ELISA plate reader, and IC50 values were calculated. Similarly, IC50 values were determined for all MEFs prepared from leaflets collected from various parts of India throughout the year. Additionally, IC50 values with other cell lines were also determined with MEF_TN_ (0–30 *μ*g/ml).

Mahanine/mahanimbine in absolute ethanol and koenimbine in DMSO were taken to make 5000 *μ*M stock for *in vitro* studies. Substocks were prepared with complete medium. PA1 and OVCAR3 were incubated separately with 0–100 *μ*M of purified mahanine, mahanimbine, and koenimbine for 48 h and processed similarly.

### 2.7. In Vivo Xenograft Mice Model

MEF_TN_ (1.0 gram) was dissolved in ethanol (6.0 ml) diluted with sterile double distilled water (34 ml) to form a uniform suspension and used for oral feeding in mice. Before the initiation of the experiment, all mice were acclimated for 7 days and were kept five per group in individually ventilated cages (IVC) and used for *in vivo* efficacy studies.

Female nude mice (4–6 weeks, *n* = 10) were injected subcutaneously with PA1 and A549 cells (7 × 10^6^) separately. Tumors (∼100 mm^3^) were generated within 10–12 days. Animals were fed orally with MEF_TN_ (300 mg/kg BW) two times per day for 31 days in ovarian and 14 days in lung cancer. Control/untreated animals were fed with vehicle (15% ethanol in water). The tumor size was monitored with screw gauge. Body weight during the treatment was also measured. This study has been approved by the institute's ethical committee on animal experimentation.

### 2.8. Statistical Analysis

The data shown were representative of three sets of independent experiments. The results were represented as mean ± SD from independent experiments.

## 3. Results and Discussion

### 3.1. Enrichment of Mahanine in *M. koenigii* Extract

Our study for more than a decade established mahanine as a therapeutic lead molecule and may be considered as a main active principle in these leaflets. Here, we have used this carbazole alkaloid as the main stable reference marker.

However, isolation of the single molecules from the plant material is generally a multistep procedure which leads to low yield. More importantly, the majority of the plant-derived molecules are stereoisomers. The plant specifically synthesizes either levo (−) or dextro (+) isomer [11]. The activity of stereoisomers is not always the same; in fact, sometimes these are antagonistic [12]. Synthesis and purification of specific stereoisomers are difficult, time-taking, and costly process. Interestingly, plant-derived (−) mahanine is levo, which is a biologically active isomer. Even after two decades of the discovery of mahanine, the synthesis of this alkaloid is very difficult even today. Very recently, Hou et al. reported the synthesis of (±) mahanine involving eight steps with lesser yield [13]. This synthetic mahanine is expected to be less active as it is a mixer of two enantiomers. Therefore, isolation or synthesizing mahanine is commercially not viable.

**Figure 1:**
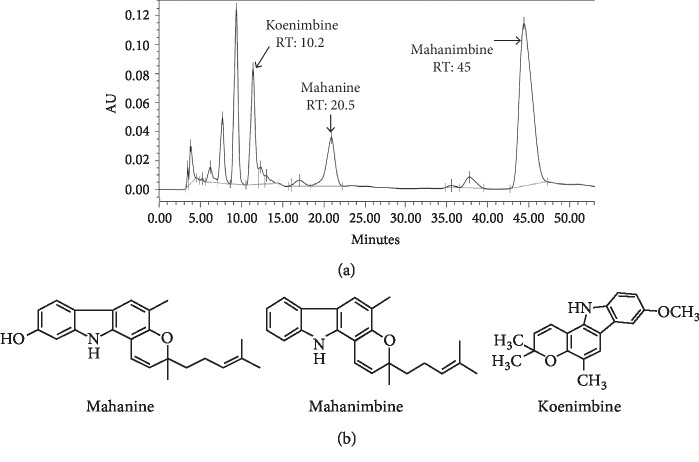
(a) HPLC profile of MEF_WB_ and structures of identified markers. The arrows indicate the peaks corresponding to the three isolated markers matched with their retention times in the HPLC profile of MEF_WB_. (b) Chemical structures of three carbazole alkaloids isolated from MEF_WB_ used as markers.

Considering all these problems, here, we have prepared a mahanine-enriched fraction (MEF) using two edible nontoxic solvents, namely, water and ethanol, within a short time involving only two steps, which makes the final product cheaper and easy to commercialize. As mahanine is insoluble in water, clean leaflets collected from West Bengal during the September–December were extracted with hot water, and we discarded the water-soluble fraction. Next, maceration of the remaining leaflet residue with ethanol was performed. HPLC analysis of 25 *µ*g of sample demonstrated (Figure 1(a)) that the ethanolic extract after removal of water-soluble fraction exhibited enhanced mahanine content (14%) (Table 2).

Each fraction was separately tested at different concentrations for its reduced metabolic activity against PA1 by the MTT assay. IC50 values were compared with the untreated control. Water extract exhibited very low/minimum activity (Table 2). The ethanolic extract, after removal of inactive water-soluble fraction, exhibited more reduction in metabolic activity, IC50 being 37 *μ*g/ml. Therefore, this fraction is named as a mahanine-enriched fraction (MEF).

### 3.2. Isolation of Marker Compounds in MEF

The major problem for herbal medicine is the batch to batch variations of chemical compositions which adversely influence its biological activity [14]. To minimize these variations, we need to have a few well-characterized chemical markers for validating HPLC fingerprinting. For this purpose, we have identified three marker molecules, namely, mahanine, mahanimbine, and koenimbine from MEF. Previously, our group reported mahanine and mahanimbine from the methanolic extract of *M. koenigii* [4].

Here, we have isolated the mahanine and mahanimbine using the reference compounds, and purity was confirmed by HPLC ([Supplementary-material supplementary-material-1]). ESI–MS ([Supplementary-material supplementary-material-1]), ^1^H ([Supplementary-material supplementary-material-1]), and ^13^C NMR spectral data analyses established their structures as mahanine and mahanimbine (Figure 1(b)). During this process, we have also isolated another carbazole alkaloid, koenimbine, and characterization was again confirmed by HPLC, ESI-MS, NMR, and compared with the available literature (PubChem CID: 97487). The calibration curves for all three molecules were plotted with three different concentrations (5, 10, and 20 *μ*g) for further studies ([Supplementary-material supplementary-material-1]; [Supplementary-material supplementary-material-1]).

### 3.3. Biological Activity of Three Carbazole Alkaloids Isolated from MEF against Ovarian Cancer Cells

We have earlier compared the degree of apoptosis between mahanine and mahanimbine in lymphoblastic leukemia, myeloid leukemia, glioma, pancreatic, lung, colorectal, and cervical cancers cells [4]. Koenimbine reduced metabolic activity of prostate cancer [15], breast cancer [16], and liver cancer cells [17]. However, the biological activity of mahanimbine and koenimbine was not tested in ovarian cancer cells. In our previous studies, we have tested mahanine-induced cell death by the MTT assay [8]. These results were corroborated with many other methods indicating reduced cell viability, i.e., apoptosis [8]. Therefore, reduced metabolic activity may indicate the antiproliferative property.

**Figure 2:**
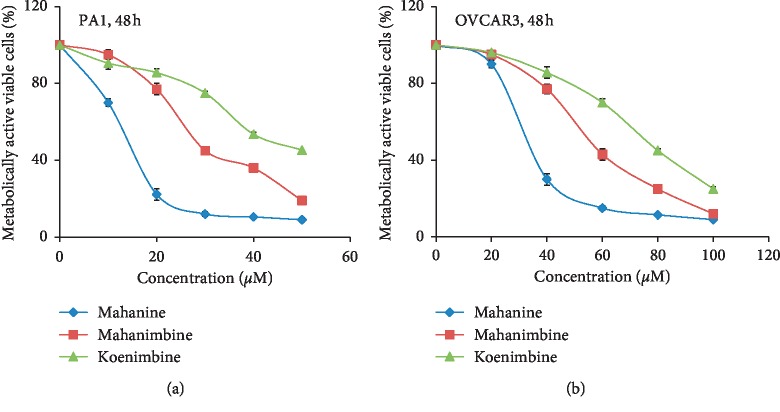
Antiproliferative activities of mahanine, mahanimbine, and koenimbine isolated from MEF_WB_ against ovarian cancer as determined by the MTT assay (a, b). Ovarian cancers cells were exposed to varying concentrations of purified mahanine, mahanimbine, and koenimbine separately for 48 h. IC50 was calculated as the percentage relative to untreated cells which were considered as 100%. Antiproliferative activity of mahanine against PA1 and OVCAR3 by MTT assay has earlier been reported [8]; here, we have repeated this experiment for comparison with mahanimbine and koenimbine. These data were derived from three individual experiments, and mean ± SD was indicated.

Here, we have compared the antiproliferative activity of all three carbazole alkaloids at different concentrations (0–100 *μ*M) both in PA1 and a cisplatin-resistant (OVCAR3) ovarian cancer cells by the MTT assay (Figures 2(a) and 2(b)). All these molecules induce cell death in a concentration-dependent manner. Mahanine exhibited the highest activity against PA1 and OVCAR3 compared to others under identical conditions (Table 3).

### 3.4. Seasonal Variations of Mahanine

**Figure 3:**
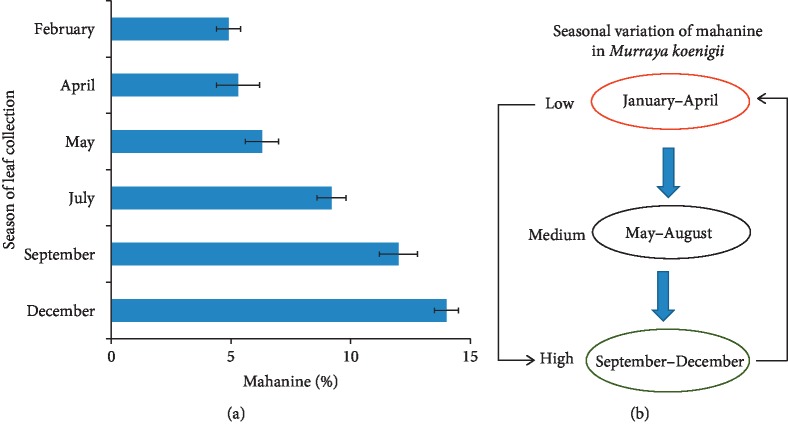
Seasonal variation of mahanine in MEF_WB_. (a) MEF_WB_ prepared from the leaflets collected during different seasons was analyzed by HPLC. The variations in percent of mahanine were plotted on the graph. (b) Schematic representation of seasonal variations of mahanine from low to high. These data were derived from three individual experiments, and mean ± SD was indicated.

Our next aim was to select the best season for collection of leaflets with a high quantity of mahanine for MEF preparation. Initially, we have collected leaflets from West Bengal for every two months throughout the year for three consecutive years. MEF was prepared (MEF_WB_) for each batch, and mahanine content was analyzed by HPLC. The highest amount of mahanine was present in leaflets collected during September to December (14 ± 1.5%), which drops to a minimum during January–April (∼5%) and medium (∼6–10%) during May–August (Table 4, Figures 3(a) and 3(b)). However, the percent of mahanimbine and koenimbine was 9–20% and 8–15%, respectively. A minimum amount of koenimbine was observed from May to July. A similar trend was observed in three years.

The IC50 of all MEFs was tested with different concentrations and compared against PA1 (Table 4). The IC50 of MEF from the leaflets collected during September–December was highest corroborating with a higher content of mahanine suggesting the best time for collection.

### 3.5. Variation of Mahanine in the Leaflets Collected from Different Geographical Locations

Chemical composition/active principle of the plant varies from place to place in a wide range from 0.9 to 100 [18]. The medicinal value of a plant is directly proportional to the quantity of biologically active principles. Mahanine is the most active carbazole alkaloid found in *M. koenigii*.

**Figure 4:**
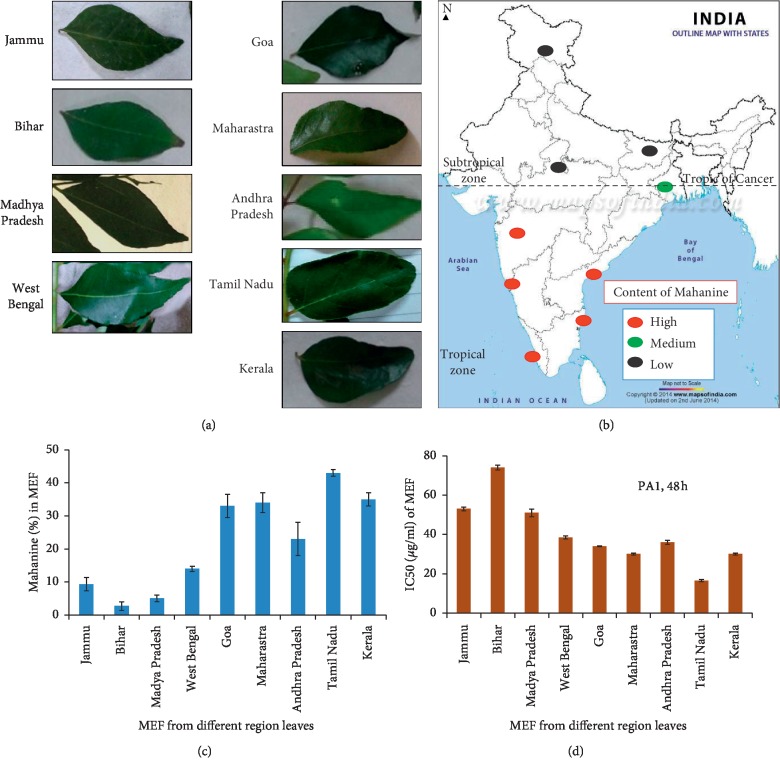
Geographical variations of mahanine, mahanimbine, and koenimbine and its relation with antiproliferative activity. (a) Morphological differences in leaflets collected from nine different locations. (b) Locations for leaflets collection were marked on the map; highlights with black, green, and red dots represent the low, medium, and high quantities of mahanine. (c) The percent of mahanine in MEFs prepared from different places. (d) IC50 values of MEFs were checked for their activity against PA1 for 48 h by the MTT assay. These results were compared with mahanine quantity as depicted in Figure 4(d). These data were derived from three individual experiments, and mean ± SD was indicated.

**Figure 5:**
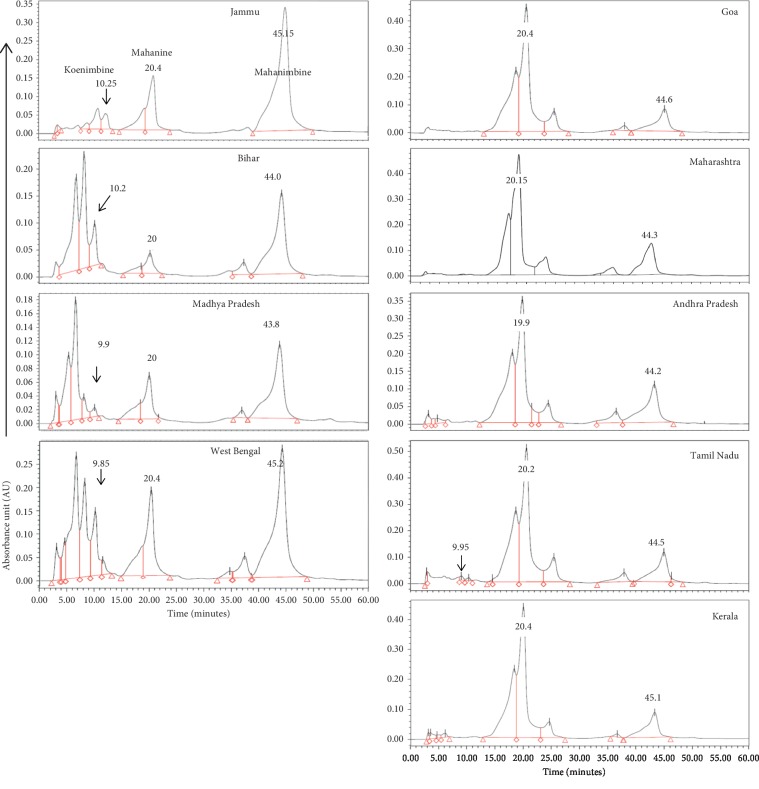
HPLC fingerprints all MEFs, showing the geographical variations of three markers. HPLC profiles of MEFs from leaflets were marked for mahanine, mahanimbine, and koenimbine with their respective retention times (RTs) for comparison.

To standardize the place, we have collected leaflets from nine different regions covering all parts of India from September to December in three consecutive years. The plant material was authenticated by DNA bar-coding as reported earlier [19]. Leaflets of different regions varied in their shape, size, thickness, color (dark/light green), and odor (Figure 4(a)). The yield (gram of MEF/100 g of the dry leaf) varied from 5.5% to 9.0%, Tamil Nadu being the highest. Interestingly, the percent of mahanine varied in a wide range from ∼2.7 to 43% (Table 5). The amount of mahanine (Figures 4(b) and 4(c)) is highest in Tamil Nadu (MEF_TN_; ∼43 ± 2.5%), and Bihar leaflets exhibited the lowest quantity (MEF_B_; 2.7 ± 1.3%). However, the percent of mahanimbine (∼8.3%) and koenimbine (∼0.8%) in MEF_TN_ was significantly low. The percent of these two alkaloids differs from place to place. In most of the places, the percent of koenimbine is either low or sometimes not even detectable. HPLC fingerprint of MEF, prepared from all these leaflets, is also different (Figure 5). The percent of alkaloids present in the leaflets collected during three years did not vary significantly.

The content of mahanimbine is inversely proportionally to the mahanine in the majority of the places, Jammu (MEF_J_) being the highest. It is worthwhile to mention that these two molecules just differed with a hydroxyl group (-OH) at C7 position. We have earlier demonstrated the structure-activity relationship of these two alkaloids and confirmed that -OH group is crucial for its antiproliferative effect against cancer cells [4].

The highest yield of MEF and amount of mahanine in these leaflets depends on various factors including temperature, rainfall, and sunshine in this Torrid Zone. In summary, the best place for collecting leaflets with the highest quantity of mahanine is southern part (tropical zone), Tamil Nadu (MEF_TN_). Our data proved that selection of the place for collection of plant material is an essential component for preparing any extract/fraction with the highest medicinal property.

### 3.6. Correlation of Mahanine Content and Antiproliferative Activity of MEFs

The MEF prepared from leaflets collected from different regions of India during September–December was checked for their biological activity against PA1 with various concentrations. IC50 varied from ∼15 to ∼75 *μ*g/ml, indicating ∼5 fold reduced biological activity of MEF_TN_ compared to MEF_B_ corroborating with the highest percent of mahanine (Figure 4(d)).

Statistical evaluation of these results by the “Spearman-rank” correlation method showed 0.97 (97.6%) which confirms the significance of the positive correlation between mahanine content in the leaflets and its biological activity against cancer cells. Therefore, the medicinal values of the plant are directly proportional to the mahanine content.

Although other two markers (mahanimbine and koenimbine) showed reduced metabolic activity separately, they present in minute amount, respectively, in MEF_TN_ (Table 5) is compared to biologically more active mahanine (∼43%). Due to the low concentration of mahanimbine and koenimbine, they may not contribute significantly to the total activity of MEF_TN_.

Since mahanine content is more in MEF_TN_; next, we have compared the percent of this alkaloid in the leaflets collected from ten different regions within the Tamil Nadu during different seasons. As expected, mahanine content is more from September to December. Leaflets from all ten places showed a good amount of mahanine varying from 35 to 45%. IC50 of MEF from these leaflets was in the range of 15–20 *μ*g/ml. Here, also we confirmed that the activity of MEF depends upon the percent of the mahanine present in these leaflets.

### 3.7. In Vitro Activity of MEF against Various Cancer Cell Lines of Different Origins

So far, our results demonstrated that both the mahanine content and biological activity are highest in MEF_TN_. Next, this (0–60 *μ*g/ml) was tested against sixteen cancer cell lines with different mutations and chemoresistant status belonging to nine different cancers. MEF reduced the metabolic activity in all these cell lines in a concentration-dependent manner. IC50 values were in the range of ∼13.2–19.0 *μ*g/ml in ovarian, lung, Grade IV glioblastoma, melanoma, pancreatic, colorectal, cervical, osteosarcoma, and oral cancers (Table 1). MEF_TN_ (0–150 *μ*g/ml) was tested against normal lung fibroblast cells, IC50 being ∼80 *μ*g/ml. This result demonstrates the potentiality of MEF as a broad antiproliferative agent in many different cancers with various mutations at low concentration.

Additionally, we observed that except OVCAR3 and UWB1.289+BRCA1, all other cells exhibited IC50 < 20 *μ*g/ml of MEF_TN_. OVCAR3 cells are reported to be resistant to cisplatin, Adriamycin, and melphalan [20]. UWB1.289+BRCA1 cells having BRCA1 mutation is resistant to PARP inhibitor drugs like rucaparib, olaparib, and veliparib [21]. Due to these inherent drug-resistance properties, these two cell lines showed higher IC50 values compared to others.

### 3.8. Efficacy of MEF_TN_ in Ovarian and Lung Cancer Xenograft Models

**Figure 6:**
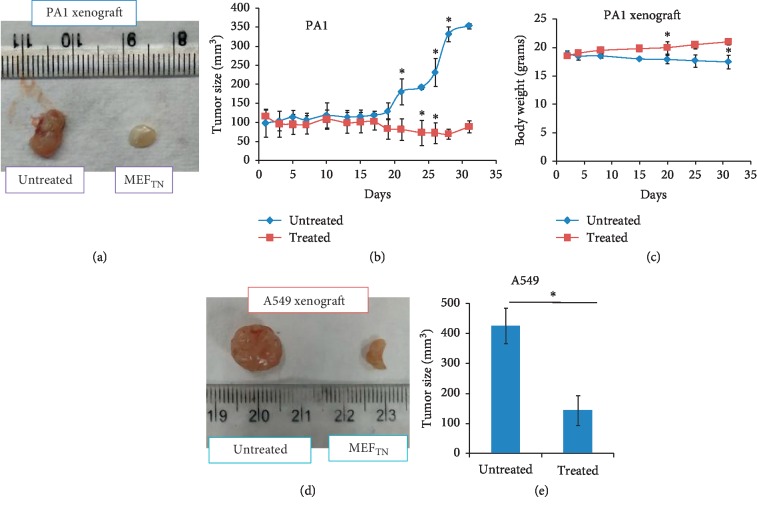
Activity of MEF_TN_ in ovarian and lung cancer xenograft model in nude mice. (a, d) Ovarian cancer and lung cancer models were generated in nude mice using PA1 and A549 cell lines, respectively. Representative images of both the tumors were presented. (b, e) MEF_TN_ was fed orally and tumor size was compared with untreated tumor-bearing mice. (c) Body weights were monitored and plotted. ^*∗*^Significant difference at *p* < 0.05.

To check the *in vivo* efficacy of MEF_TN_, we have used two representative ovarian and lung cancers models. MEF_TN_-treated mice (300 mg/kg BW *p.o.* BID) showed reduction in tumor size from ∼354 to 89 mm^3^ in ovarian cancer compared to the untreated animals (Figures 6(a) and 6(b) and Table 6). No weight loss was observed in MEF-treated tumor-bearing mice up to 31 days indicating no apparent toxicity in other organs (Figure 6(c)). Similarly, MEF_TN_-treated animal exhibited a reduced tumor mass from ∼426 to 170 mm^3^ in lung cancer model (Figures 6(d) and 6(e)).

## 4. Conclusions

**Figure 7:**
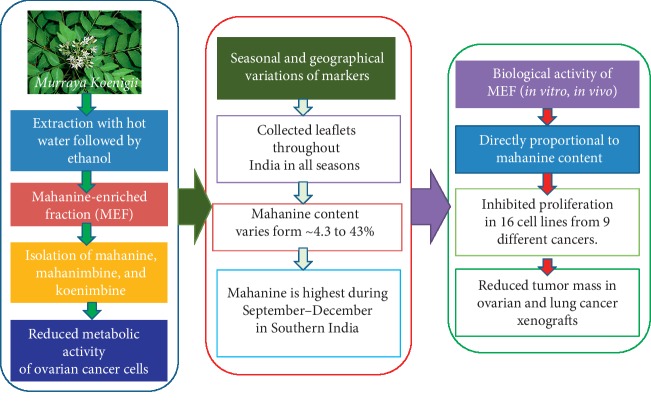
Summary of the study. This figure shows the workflow and conclusions of the study. These data were derived from three individual experiments, and mean ± SD was indicated. ^*∗*^The significant difference of *p* < 0.05.

We are reporting a mahanine-enriched fraction (MEF) prepared using two nontoxic edible solvents in two steps. Three carbazole alkaloids, namely, mahanine, mahanimbine, and koenimbine, have been isolated and characterized. This is the first report showing reduced metabolic activity of mahanimbine and koenimbine in ovarian cancer cells in a concentration-dependent manner. Our systematic study (Figure 7) reveals that the percent of mahanine is highest in leaflets collected from Tamil Nadu (MEF_TN_) from September to December. MEF_TN_ exhibited the highest activity against cancer cells. Thus, the medicinal values of this plant are directly proportional to the amount of mahanine present in the leaflets. Most importantly, MEF_TN_ significantly reduces tumor mass both in ovarian and lung cancer xenograft models. Taken together, this study suggests that we can improve the medicinal property of this high-value plant to ∼5 fold by right selection of season and place for leaf collection. MEF_TN_ also showed reduced metabolic activity in sixteen cells lines from nine different origins in a concentration-dependent manner. To the best of our knowledge, this is the first report demonstrating variations of mahanine, mahanimbine, and koenimbine in this important herb collected during different seasons from various locations of India reflected in their medicinal values. Such a study is expected to increase the commercial values of this medicinal plant and enhance the efficacy of phytopharmaceuticals prepared from this herb.

## Figures and Tables

**Table 1:** Biological activity of MEF_TN_.

S. No.	Cancer type	Cell lines	Mutation/drug-resistant	Culturing media + 10% FBS	MEF_TN_ IC50 (*μ*g/ml)
1	Ovarian	PA1	NRAS mutation anoikis-resistant	MEM	15 ± 0.2
2		OVCAR3	TP53 mutation drug-resistant	RPMI 1640	27 ± 0.18
3		SKOV3	TP53 mutation	McCoy's 5a	16.5 ± 0.11
4		OAW42	ARID1A, PIK3CA mutation	DMEM	15.5 ± 0.05
5		UWB1.289 + BRCA1	BRCA1 mutation	MEBM + RPMI 1640 (1 : 1)	25 ± 0.2

6	Lung carcinoma	A549	KRAS, CDKN2A mutation	IMDM	13.0 ± 0.09

7	Grade IV glioblastoma	U-87 MG	PTEN mutation	IMDM	16.2 ± 0.15
8		U87MG vIII	EGFR mutation	IMDM	18.7 ± 0.02

9	Pancreatic carcinoma	MIA PaCa-2	TP53, K-RAS mutation, p16 deletion	RPMI-1640	14.1 ± 0.1

10	Colorectal	HCT116	KRAS, PIK3CA mutation	IMDM	13.4 ± 0.4

11	Cervical	Hela	STK11, CTNNB1, CNTN1 mutations	IMDM	15.45 ± 0.05

12	Squamous cell oral carcinoma	UPCI-SCC084	TP53 mutation	DMEM	14.5 ± 0.25
13		UPCI-SCC131	TP53 wild type	DMEM	15.5 ± 0.25

14	Osteosarcoma	MG63	CDKN2A mutation	MEM	14.9 ± 0.22

15	Melanoma	ChaMel47	No reports on mutation	IMDM	18.5 ± 0.21
16		Sk-mel-28	B-RAF, TP53 mutation	RPMI 1640	19 ± 0.11

17	Normal lung fibroblast cells	WI-38	—	MEM	>80 ± 0.8

Antiproliferative activity of mahanine-enriched fraction prepared from Tamil Nadu leaflets (MEF_TN_) was analyzed by the MTT assay. IC50 values and mutation status of all cancers cell lines were tabulated. These data were derived from three individual experiments, and mean ± SD was indicated.

**Table 2:** Preparation of MEF form *M. koenigii* leaflets from West Bengal.

Extract	Mahanine (%)	IC50 (*μ*g/ml)
Water extract	0	90
Mahanine-enriched fraction (MEF_WB_)	14	37

Comparison of the water extract and MEF_WB_ for their mahanine content and biological activity against PA1. IC50: the half-maximal inhibitory concentration.

**Table 3:** Reduced metabolic activity by three carbazole alkaloids from MEF against ovarian cancer.

Ovarian cancer cell lines	IC50 (*μ*M)		
	Mahanine	Mahanimbine	Koenimbine
PA1	11 ± 0.2	28 ± 0.11	51 ± 0.25
OVCAR3	32 ± 0.18	52 ± 0.05	68 ± 0.22

Cells were treated separately with mahanimbine and koenimbine for 48 h, and mahanine was used as the control for comparison [8]. IC50 values were compared. These data were derived from three individual experiments, and mean ± SD was indicated.

**Table 4:** Seasonal variation of three markers and antiproliferative activity of MEF_WB_.

The month of collection of leaflets	Mahanine (%)	Mahanimbine (%)	Koenimbine (%)	MEF_WB_ IC50 (*μ*g/ml)
February	5 ± 1.5	12 ± 1.4	15 ± 1.8	73.2 ± 0.15
April	5 ± 1.9	11 ± 1.8	12 ± 1.2	71.0 ± 0.5
May	6 ± 1.7	9 ± 1.5	8 ± 0.9	68.0 ± 0.14
July	9 ± 1.6	16 ± 1.2	8 ± 1.5	55.0 ± 0.3
September	12 ± 1.8	18 ± 1.6	10 ± 1.1	39.0 ± 0.3
December	14 ± 1.5	20 ± 1.6	11 ± 1.25	37.0 ± 0.2

Composition of markers in MEF_WB_ in different seasons and their IC50. These are representative data collected in one year. MEF_WB_: mahanine-enriched fraction prepared from West Bengal leaflets. These data were derived from three individual experiments, and mean ± SD was indicated.

**Table 5:** Regional variation of mahanine, mahanimbine, and koenimbine in different MEFs.

Region of leaf collection	Yield: a gram of MEF/100 g of dry leaf	Mahanine (%)	Mahanimbine (%)	Koenimbine (%)
Jammu (MEF_J_)	6.4 ± 0.5	9.3 ± 1.6	27.0 ± 2.5	6.3 ± 1.2
Bihar (MEF_B_)	5.8 ± 0.2	2.7 ± 1.3	12.0 ± 1.5	7.3 ± 1.4
Madhya Pradesh (MEF_MP_)	7.2 ± 0.1	5.0 ± 0.9	8.6 ± 0.9	1.2 ± 0.9
West Bengal (MEF_WB_)	5.5 ± 0.5	14.0 ± 0.8	20.7 ± 1.2	11.9 ± 1.1
Goa (MEF_G_)	6.5 ± 0.6	33.0 ± 2.9	6.3 ± 0.9	ND
Maharashtra (MEF_M_)	5.9 ± 0.1	34.0 ± 2.7	10.0 ± 1.1	ND
Andhra Pradesh (MEF_AP_)	5.9 ± 0.25	23.0 ± 4.1	9.2 ± 0.9	ND
Tamil Nadu (MEF_TN_)	9.0 ± 0.7	43.0 ± 2.5	8.3 ± 1.7	0.8 ± 0.3
Kerala (MEF_K_)	8.0 ± 0.5	35 ± 4.0	6.3 ± 1.2	ND

The yield of all MEFs (mahanine-enriched fractions) and their markers as obtained from HPLC. ND: not detected. A representative profile of these alkaloids in leaflet collected during one year has been tabulated. These data were derived from three individual experiments, and mean ± SD was indicated.

**Table 6:** *In vivo* efficacy of MEF_TN_ in tumor xenograft models.

Groups	Ovarian (PA1)	Lung (A549)
	Tumor size (mm^3^) after the treatment period	
Untreated	354 ± 7.1	426 ± 60
MEF_TN_	89 ± 15	170 ± 43

Xenograft models were generated in nude mice. MEF_TN_ (300 mg/kg BW *p.o.* BID) was fed orally in treated groups, and vehicle was fed to the untreated group for 31 days in ovarian and 14 days in lung cancer. Mean ± SD was indicated in the result.

## Data Availability

No data were used to support this study.

## Abbreviations

OC: Ovarian cancer
MEF: Mahanine-enriched fraction
MEF_WB_: MEF from West Bengal leaflets
MEF_TN_: MEF from Tamil Nadu leaflets
MEF_B_: MEF from Bihar leaflets
IC50: The half-maximal inhibitory concentration
BW: Bodyweight
MTT: 3-(4,5-Dimethylthiazol-2-yl)-2,5-diphenyltetrazolium bromide
FBS: Fetal bovine serum
*p.o.*: Per os (oral administration)
BID: Bis in die (twice daily).

## Supplementary Materials

Supplementary Materials Figure S1: (HPLC analysis) the purity of three markers isolated from MEF and the retention time (RT) was confirmed by HPLC. Mahanine, mahanimbine, and koenimbine were dissolved in running solvent (methanol : water, 80 : 20) and filtered through 0.22 *μ*m and injected into the HPLC rheodyne injector. All the three markers were found to be pure (≥95%), and RT matched with reference compounds. Figure S2: Determination of molecular weights by electrospray ionization-mass spectrometry (ESI-MS); ESI-MS revealed that the molecular weight of mahanine (M + Na = 370.13) is 347.45, mahanimbine (M + 1 = 332.30) is 331.45, and koenimbine (M + 1 = 294.32) is 293.36. Figure S3: Confirmation of molecular structures of mahanine, mahanimbine, and koenimbine by ^1^H NMR. Mahanine: ^1^H NMR (400 MHz, Methanol-*d*_4_) *δ* 7.62 (d, *J* = 7.7 Hz), 7.52, 7.45, 6.77, 6.58, 5.64–5.57 (m), 5.08, 3.28, 2.24, 2.12 (d, *J* = 6.2 Hz), 1.67 (d, *J* = 9.5 Hz), 1.60, 1.53, 1.37. Mahanimbine: ^1^H NMR (600 MHz, chloroform-*d*) *δ* 7.89 (d, *J* = 6.0 Hz, 1H), 7.82 (s, 1H), 7.64 (s, 1H), 7.31–7.26 (m, 2H), 7.16 (ddd, *J* = 6.0 Hz, 1H), 6.54 (d, *J* = 6.0 Hz, 1H), 5.58 (d, *J* = 6.0 Hz, 1H), 5.10 (ddt, *J* = 6.0 Hz, 1H), 2.32 (d, *J* = 6.0 Hz, 3H), 2.23–2.10 (m, 3H), 1.74 (dd, *J* = 9.3, 7.4 Hz, 2H), 1.65 (d, *J* = 1.7 Hz, 3H), 1.57 (d, *J* = 1.6 Hz, 3H), 1.42 (s, 3H). Koenimbine: ^1^H NMR (600 MHz, chloroform-*d*) *δ* 7.72 (s, 1H), 7.62 (s, 1H), 7.40 (d, *J* = 2.5 Hz, 1H), 7.25 (d, *J* = 1.3 Hz, 1H), 6.93 (dd, *J* = 8.7, 2.5 Hz, 1H), 6.57 (d, *J* = 9.7 Hz, 1H), 5.66 (d, *J* = 9.7 Hz, 1H), 3.90 (s, 3H), 2.32 (d, *J* = 0.9 Hz, 3H), 1.48 (s, 6H). All these NMR's were matched with the available literature (PubChem CID: 375151, 167963, and 97487, respectively). Figure S4: (calibration curves of mahanine, mahanimbine, and koenimbine) all three carbazole alkaloids were dissolved separately in methanol : water (80 : 20) and filtered for HPLC analysis. Each molecule was injected separately in three different concentrations (5, 10, and 20 *μ*g). Area vs. concentration was plotted. This calibration curve was used to calculate the amount of these markers in different MEFs. Table S1: the obtained area of each concentration for the respective molecule from Figure S4 was tabulated to calculate the amount of all three markers in different MEF's.
