# Supplementary material for: Influence of Geographical and Seasonal Variations on Carbazole Alkaloids Distribution in Murraya koenigii: Deciding Factor of Its In Vitro and In Vivo Efficacies against Cancer Cells
Source: Biomed Res Int. 2020 Feb 11;2020:7821913. doi: 10.1155/2020/7821913 (PMC7036096; doi:10.1155/2020/7821913)
Supplement: Supplementary Materials — Figure S1: (HPLC analysis) the purity of three markers isolated from MEF and the retention time (RT) was confirmed by HPLC. Mahanine, mahanimbine, and koenimbine were dissolved in running solvent (methanol : water, 80 : 20) and filtered through 0.22 μm and injected into the HPLC rheodyne injector. All the three markers were found to be pure (≥95%), and RT matched with reference compounds. Figure S2: Determination of molecular weights by electrospray ionization-mass spectrometry (ESI-MS); ESI-MS revealed that the molecular weight of mahanine (M + Na = 370.13) is 347.45, mahanimbine (M + 1 = 332.30) is 331.45, and koenimbine (M + 1 = 294.32) is 293.36. Figure S3: Confirmation of molecular structures of mahanine, mahanimbine, and koenimbine by 1H NMR. Mahanine: 1H NMR (400 MHz, Methanol-d4) δ 7.62 (d, J = 7.7 Hz), 7.52, 7.45, 6.77, 6.58, 5.64–5.57 (m), 5.08, 3.28, 2.24, 2.12 (d, J = 6.2 Hz), 1.67 (d, J = 9.5 Hz), 1.60, 1.53, 1.37. Mahanimbine: 1H NMR (600 MHz, chloroform-d) δ 7.89 (d, J = 6.0 Hz, 1H), 7.82 (s, 1H), 7.64 (s, 1H), 7.31–7.26 (m, 2H), 7.16 (ddd, J = 6.0 Hz, 1H), 6.54 (d, J = 6.0 Hz, 1H), 5.58 (d, J = 6.0 Hz, 1H), 5.10 (ddt, J = 6.0 Hz, 1H), 2.32 (d, J = 6.0 Hz, 3H), 2.23–2.10 (m, 3H), 1.74 (dd, J = 9.3, 7.4 Hz, 2H), 1.65 (d, J = 1.7 Hz, 3H), 1.57 (d, J = 1.6 Hz, 3H), 1.42 (s, 3H). Koenimbine: 1H NMR (600 MHz, chloroform-d) δ 7.72 (s, 1H), 7.62 (s, 1H), 7.40 (d, J = 2.5 Hz, 1H), 7.25 (d, J = 1.3 Hz, 1H), 6.93 (dd, J = 8.7, 2.5 Hz, 1H), 6.57 (d, J = 9.7 Hz, 1H), 5.66 (d, J = 9.7 Hz, 1H), 3.90 (s, 3H), 2.32 (d, J = 0.9 Hz, 3H), 1.48 (s, 6H). All these NMR's were matched with the available literature (PubChem CID: 375151, 167963, and 97487, respectively). Figure S4: (calibration curves of mahanine, mahanimbine, and koenimbine) all three carbazole alkaloids were dissolved separately in methanol : water (80 : 20) and filtered for HPLC analysis. Each molecule was injected separately in three different concentrations (5, 10, and 20 μg). Area vs. concentration was [file 7821913.f1.docx]

**Figure S1 HPLC analysis**

The purity of three markers isolated from MEF and the retention time (RT) was confirmed by HPLC. Mahanine, mahanimbine and koenimbine were dissolved in running solvent (methanol: water, 80:20) and filtered through 0.22 μm and injected into HPLC rheodyne injector. All the three markers were found to be pure (≥95%) and RT matched with reference compounds.

**Figure S2 Determination of molecular weights by Electron spray ionization-mass spectrometry (ESI-MS)**

ESI-MS revealed the molecular weight of mahanine (M+Na = 370.13) is 347.45, mahanimbine (M+1= 332.30) is 331.45 and koenimbine (M+1= 294.32) is 293.36

**Figure S**3 **Confirmation of molecular structures of mahanine, mahanimbine and koenimbine by** ^1^H **NMR**

**Mahanine:** ^1^H NMR (400 MHz, Methanol-*d*_4_) δ 7.62 (d, *J* = 7.7 Hz), 7.52, 7.45, 6.77, 6.58 , 5.64 – 5.57 (m), 5.08, 3.28 , 2.24 , 2.12 (d, *J* = 6.2 Hz), 1.67 (d, *J* = 9.5 Hz), 1.60 , 1.53 , 1.37.

**Mahanimbine**: ^1^H NMR (600 MHz, Chloroform-*d*) δ 7.89 (d, *J* = 6.0 Hz, 1H), 7.82 (s, 1H), 7.64 (s, 1H), 7.31 – 7.26 (m, 2H), 7.16 (ddd, *J* = 6.0 Hz, 1H), 6.54 (d, *J* = 6.0 Hz, 1H), 5.58 (d, *J* = 6.0 Hz, 1H), 5.10 (ddt, *J* = 6.0 Hz, 1H), 2.32 (d, *J* = 6.0 Hz, 3H), 2.23 – 2.10 (m, 3H), 1.74 (dd, *J* = 9.3, 7.4 Hz, 2H), 1.65 (d, *J* = 1.7 Hz, 3H), 1.57 (d, *J* = 1.6 Hz, 3H), 1.42 (s, 3H).

**Koenimbine:** ^1^H NMR (600 MHz, Chloroform-*d*) δ 7.72 (s, 1H), 7.62 (s, 1H), 7.40 (d, *J* = 2.5 Hz, 1H), 7.25 (d, *J* = 1.3 Hz, 1H), 6.93 (dd, *J* = 8.7, 2.5 Hz, 1H), 6.57 (d, *J* = 9.7 Hz, 1H), 5.66 (d, *J* = 9.7 Hz, 1H), 3.90 (s, 3H), 2.32 (d, *J* = 0.9 Hz, 3H), 1.48 (s, 6H).

All these NMR’s were matched with available literature (PubChem CID: 375151, 167963 and 97487 respectively).

**Figure S**4 **calibration curves of mahanine, mahanimbine and koenimbine**

All three carbazole alkaloids were dissolved separately in methanol: water (80:20) and filtered for HPLC analysis. Each molecule was injected separately in three different concentrations (5, 10, 20 μg). Area vs. concentration was plotted. This calibration curve was used to calculate the amount of these markers in different MEF's.

**Table-S1**

| **Mahanine/ Mahanimbine/ Koenimbine (µg)** | **Mahanine**  **Area (AU)** | **Mahanimbine**  **Area (AU)** | **Koenimbine**  **Area (AU)** |
| --- | --- | --- | --- |
| 5 | 4498720 | 4436741 | 4981180 |
| 10 | 9197521 | 8173482 | 9762360 |
| 20 | 17394042 | 16446964 | 17524720 |

*The obtained area of each concentration for the respective molecule from Fig. S4 were tabulated to calculate the amount of all three markers in different MEF's.*
